# Phase I study of A166, an antibody‒drug conjugate in advanced HER2-expressing solid tumours

**DOI:** 10.1038/s41523-023-00522-5

**Published:** 2023-04-18

**Authors:** Jian Zhang, Rujiao Liu, Shuiping Gao, Wenhua Li, Yang Chen, Yanchun Meng, Chang Liu, Wenyue Jin, Junyan Wu, Ying Wang, Yanrong Hao, Shuli Yi, Yan Qing, Junyou Ge, Xichun Hu

**Affiliations:** 1 Department of Oncology, Shanghai Medical College, Fudan University, Shanghai, 200032 P.R. China; 2 Phase I Clinical Trial Center, Fudan University Shanghai Cancer Center, Shanghai, 200032 P.R. China; 3 Department of Digestive Oncology, Fudan University Shanghai Cancer Center, Shanghai, 200032 P.R. China; 4 Breast Tumour Center, Sun Yat-sen Memorial Hospital, Guangzhou, 510120 P.R. China; 5 Guangxi Zhuang Autonomous Region People’s Hospital, Nanning, 530016 P.R. China; 6 Sichuan Kelun-Biotech Biopharmaceutical Co., Ltd, Chengdu, 611130 P.R. China; 7 Department of Breast and Urinary Oncology, Fudan University Shanghai Cancer Center, Shanghai, 200032 P.R. China

**Keywords:** Breast cancer, Drug development

## Abstract

In this phase I study, the safety, pharmacokinetics, and antitumour activity of the HER2-targeted antibody–drug conjugate A166 were evaluated in patients with HER2-expressing advanced solid tumours. Patients with advanced solid tumours refractory to standard therapies received A166 at doses of 0.1, 0.3, 0.6, 1.2, 2.4, 3.6, 4.8 or 6.0 mg/kg Q3W in a standard “3 + 3” design. Dose cohorts were expanded at 4.8 and 6.0 mg/kg Q3W. Primary endpoints were assessment of the safety and tolerability of A166 and identification of the maximum tolerated dose or recommended phase II dose. In total, 81 patients were enroled and received A166 (*n* = 1 for 0.1 mg/kg; *n* = 3 for each of 0.3, 0.6, 1.2, 2.4 and 3.6 mg/kg doses; *n* = 27 for 4.8 mg/kg; *n* = 38 for 6.0 mg/kg). No dose-limiting toxicity or drug-related deaths occurred. The most common treatment-related adverse events at grade 3 or higher were corneal epitheliopathy (30.9%), blurred vision (18.5%), dry eyes (7.4%), and peripheral sensory neuropathy (6.2%). The C_max_ and area under the curve of Duo-5, its free payload, were approximately 0.1% and 0.2% of those of the ADC, respectively. For all assessable HER2-positive breast cancer patients enroled in the 4.8 mg/kg and 6.0 mg/kg cohorts, the corresponding ORRs were 73.9% (17/23) and 68.6% (24/35), respectively, and the median PFS was 12.3 and 9.4 months, respectively. A166 has a recommended phase II dose of 4.8 mg/kg Q3W, manageable toxicity, good stability in the circulation and promising antitumour activities in HER2-positive breast cancer patients.

## Introduction

Human epidermal growth factor receptor 2 (HER2) is a member of the epidermal growth factor receptor family that contributes to tumour cell proliferation, adhesion, migration, differentiation, and apoptosis^1^. The overexpression or amplification of HER2-negatively impacts the survival of patients with both early and advanced disease^1–3^. Biologicals targeting HER2 are the standard of care in the treatment of cancers, such as breast and gastric cancers. The introduction of HER2-targeted therapies, most notably trastuzumab, pertuzumab, antibody‒drug conjugates (ADCs; e.g., trastuzumab emtansine [T-DM1] and trastuzumab deruxtecan [T-DXd; formerly DS-8201]), and tyrosine kinase inhibitors (TKIs; e.g., lapatinib, neratinib, pyrotinib and tucatinib) has led to dramatic improvements in the prognosis of patients with HER2-positive breast cancer^4^. Chemotherapy plus trastuzumab is also the recommended first-line therapy according to the ToGA trial^5,6^. Moreover, growing evidence justifies the application of anti-HER2 therapy in HER2-positive colorectal cancer^7,8^.

ADCs are one of the fastest growing anticancer drugs. T-DM1 is a second-line option for HER2-positive advanced breast cancer with an objective response rate (ORR) of 43.6% and a median progression-free survival (PFS) of approximately 9.6 months^9^. T-DXd, a third-generation ADC, achieved superior PFS and ORR over T-DM1 in a phase III study (Destiny-Breast 03) and has become a new standard of care for second-line therapy^10^. As an optimal third- or later-line therapy, T-DXd was effective in T-DM1-resistant or T-DM1-refractory patients and achieved a tumour response rate of 61.4% and a median PFS of 19.4 months in the pivotal phase II HER2-positive metastatic breast cancer trial^11^. Despite these advances, there are still unmet needs for continuous blockade with new HER2-targeted agents, since advanced breast cancer is still an incurable disease and there is no standard of care for patients who have received two lines of anti-HER2 regimens in China and those who have received T-DXd globally.

A166 is a HER2-targeted ADC composed of a cytotoxic drug (Duostatin-5 [Duo-5], anti-microtubule agent) with site-specific conjugation to a humanized anti-HER2 antibody via a stable protease-cleavable valine citrulline linker. The anti-HER2 antibody component has the same amino acid sequence as trastuzumab. The unique linker is stable in plasma and selectively cleaved by lysosomal cathepsins that are upregulated in cancer cells, which effectively prevents the premature release of toxin molecules outside tumour cells and reduces systemic toxicity^12^. A166 has a drug­to­antibody ratio (DAR) of two with homogeneous conjugation, enabling delivery of a high activity payload to targeted cells, and exhibited better tumour growth inhibition than T-DM1 at a dose of 3 mg/kg in xenograft models^13^. These unique properties make A166 a more optimized anti-HER2 agent. The results from its first-in-human study in the US (ClinicalTrials.gov number, NCT03602079) showed preliminary antitumour activity in patients with relapsed or refractory advanced HER2-altered solid cancers, mostly nonbreast cancers (i.e., HER2-positive gastric cancer, HER2-expressing ovarian cancer, HER2-mutated non-small cell lung cancer), with objective responses occurring in 36% of the patients at dose levels of 3.6 mg/kg and 4.8 mg/kg^14^.

According to regulatory requirements, we simultaneously conducted an independent single-arm phase I study in China to determine the safety, tolerability, pharmacokinetics, and clinical activity of A166 in Chinese patients with HER2-expressing locally advanced or metastatic solid tumours. Overall, the study demonstrates that A166 has manageable toxicity, good stability in circulation, and promising antitumour activities in HER2-positive breast cancer patients.

## Results

### Patient disposition and baseline characteristics

In total, 81 patients with advanced solid tumours were enroled in the present study between August 1, 2018, and May 13, 2021. Patient demographics and baseline characteristics of the study population are summarized in Table 1. Breast, colorectal, and gastric or gastroesophageal junction cancers were present in 90.1% (73/81), 7.4% (6/81), and 2.5% (2/81) of patients, respectively. HER2 expression was available for all 81 patients: 64.2% (52/81) were immunohistochemistry (IHC) 3+, 24.7% (20/81) were IHC 2+ and FISH positive, 2.5% (2/81) were IHC2+ but FISH negative, and 8.6% (7/81) were IHC 1+. Hormone receptors were evaluated in 73 breast cancer patients: 41.1% (30/73) were oestrogen receptor (ER) or progesterone receptor (PR) positive, and 58.9% (43/73) were ER and PR negative.

**Table 1:** Baseline demographic and clinical characteristics.

Characteristics No. of patients (%)	0.1 mg/kg (*n* = 1)	0.3 mg/kg (*n* = 3)	0.6 mg/kg (*n* = 3)	1.2 mg/kg (*n* = 3)	2.4 mg/kg (*n* = 3)	3.6 mg/kg (*n* = 3)	4.8 mg/kg (*n* = 27)	6.0 mg/kg (*n* = 38)	Total (*n* = 81)
Median age, years (range)	47	63 (52, 64)	52 (51, 59)	49 (39, 60)	52 (50, 67)	64 (61, 74)	53 (33, 67)	49.5 (26, 72)	52 (26, 74)
*Sex*									
Female	1 (100)	3 (100)	3 (100)	2 (66.7)	0	2 (66.7)	25 (92.6)	38 (100)	74 (91.4)
Male	0	0	0	1 (33.3)	3 (100)	1 (33.3)	2 (7.4)	0	7 (8.6)
*Cancer type*									
Breast	1 (100)	3 (100)	3 (100)	2 (66.7)	0	2 (66.7)	24 (88.9)	38 (100)	73 (90.1)
Stomach or gastroesophageal junction	0	0	0	0	1 (33.3)	1 (33.3)	0	0	2 (2.5)
Colorectum	0	0	0	1 (33.3)	2 (66.7)	0	3 (11.1)	0	6 (7.4)
*HER2 expression (IHC)*									
1+	1 (100)	0	1 (33.3)	0	1 (33.3)	0	1 (3.7)	3 (7.9)	7 (8.6)
2 + (ISH positive)	0	2 (66.7)	2 (66.7)	0	0	1 (33.3)	5 (18.5)	10 (26.3)	20 (24.7)
2 + (ISH negative or NE)	0	0	0	2 (66.7)	0	0	0	0	2 (2.5)
3+	0	1 (33.3)	0	1 (33.3)	2 (66.7)	2 (66.7)	21 (77.8)	25 (65.8)	52 (64.2)
*Hormone receptor*^a^									
ER or PR positive	0	2 (66.7)	2 (66.7)	2 (100)	0	0	11 (45.8)	13 (34.2)	30 (41.1)
ER and PR negative	1 (100)	1 (33.3)	1 (33.3)	0	0	2 (100)	13 (54.2)	25 (65.8)	43 (58.9)
*Sites of metastatic disease*									
Brain	0	0	0	0	0	0	1 (3.7)	1 (2.6)	2 (2.5)
Bone	0	1 (33.3)	1 (33.3)	1 (33.3)	0	1 (33.3)	14 (51.9)	18 (47.4)	36 (44.4)
Lung	1 (100)	2 (66.7)	2 (66.7)	2 (66.7)	2 (66.7)	2 (66.7)	17 (63.0)	17 (44.7)	45 (55.6)
Liver	0	1 (33.3)	3 (100)	2 (66.7)	3 (100)	0	12 (44.4)	15 (39.5)	36 (44.4)
Lymph node	1 (100)	1 (33.3)	1 (33.3)	0	1 (33.3)	3 (100)	11 (40.7)	21 (55.3)	39 (48.1)
*Prior treatment lines in the metastatic setting*									
<3	0	1 (33.3)	1 (33.3)	0	0	0	1 (3.7)	5 (13.2)	8 (9.9)
≥3	1 (100)	2 (66.7)	2 (66.7)	3 (100)	3 (100)	3 (100)	26 (96.3)	33 (86.8)	73 (90.1)

*ER* oestrogen receptor, *PR* progesterone receptor, *NE* not evaluable, *ADC* antibody drug conjugate, *TKI* tyrosine kinase inhibitor.

^a^For 73 breast cancer patients.

The dose escalation set included 22 patients: one at 0.1 mg/kg and three at 0.3, 0.6, 1.2, 2.4, 3.6, 4.8, and 6.0 mg/kg dose levels. The dose expansion set included 59 patients: 24 at 4.8 mg/kg and 35 at 6.0 mg/kg. All 81 patients were evaluable for toxicity analysis, whereas 80 were evaluable for tumour response. At the time of the data analysis on July 13, 2022, A166 treatment was discontinued in 68 (84.0%) of the 81 patients, most commonly due to progressive disease (62 patients) but also because of treatment-related AEs (TRAEs) (5 patients) and protocol deviation (1 patient).

Among all 81 patients, 90.1% (73/81) had received at least 3 prior lines of systemic therapy in the metastatic setting. Common treatment regimens included anti-HER2 and endocrine therapy, which was used in 95.9% (70/73) and 41.1% (30/73) of breast cancer patients, respectively. For 66 HER2-positive (IHC 2+ and FISH+ or IHC 3+) breast cancer patients, all had prior HER2-targeted therapy with a median prior line of 4, including 100% (66/66) who received trastuzumab, 89.4% (59/66) who received anti-HER2 TKIs, 28.8% (19/66) who received pertuzumab, and 25.8% (17/66) who received anti-HER2 ADCs, among whom 11 received T-DM1, 5 received ARX-788^15^, and 1 received TAA013.

### Safety

All 81 patients received at least one dose of A166 and were included in the safety analysis. No DLTs were observed in the dose escalation part; thus, the maximum tolerated dose (MTD) was not reached. TRAEs of any grade were documented in 98.8% (80/81) of patients (Table 2). Across all dose levels, the most frequent TRAEs of any grade were corneal epitheliopathy (84.0%), blurred vision (74.1%), peripheral sensory neuropathy (53.1%), dry eyes (32.1%), muscular weakness (28.4%), anaemia (23.5%), increased blood creatine phosphokinase (22.2%), alopecia (22.2%), increased alanine aminotransferase (ALT) (18.5%), increased aspartate aminotransferase (AST) (18.5%), increased myoglobin blood (17.3%), hyponatremia (16.0%), and hypomagnesemia (16.0%) (Table 2). TRAEs grading ≥3 occurred in 40 patients (49.4%) and included corneal epitheliopathy (30.9%), blurred vision (18.5%), dry eyes (7.4%), peripheral sensory neuropathy (6.2%), anaemia (2.5%), hyponatremia (2.5%), muscular weakness (2.5%), and leucopenia (1.2%).

**Table 2:** Most common TRAEs (any and grade ≥3) that occurred in 10% or more patients.

TRAEs No. of patients (%)	≤2.4 mg/kg (*N* = 13)		3.6 mg/kg (*N* = 3)		4.8 mg/kg (*N* = 27)		6.0 mg/kg (*N* = 38)		Total (*N* = 81)	
	Total	Grade ≥ 3	Total	Grade ≥ 3	Total	Grade ≥ 3	Total	Grade ≥ 3	Total	Grade ≥ 3
Overall	12 (92.3)	3 (23.1)	3 (100)	2 (66.7)	27 (100)	14 (51.9)	38 (100)	21 (55.3)	80 (98.8)	40 (49.4)
Corneal epitheliopathy	1 (7.7)	0	3 (100)	1 (33.3)	27 (100)	11 (40.7)	37 (97.4)	13 (34.2)	68 (84.0)	25 (30.9)
Blurred vision	0	0	1 (33.3)	0	23 (85.2)	7 (25.9)	36 (94.7)	8 (21.1)	60 (74.1)	15 (18.5)
Peripheral sensory neuropathy	1 (7.7)	0	0	0	17 (63.0)	1 (3.7)	25 (65.8)	4 (10.5)	43 (53.1)	5 (6.2)
Dry eyes	0	0	3 (100)	0	9 (33.3)	3 (11.1)	14 (36.8)	3 (7.9)	26 (32.1)	6 (7.4)
Muscular weakness	0	0	0	0	13 (48.1)	1 (3.7)	10 (26.3)	1 (2.6)	23 (28.4)	2 (2.5)
Anaemia	3 (23.1)	1 (7.7)	1 (33.3)	0	4 (14.8)	0	11 (28.9)	1 (2.6)	19 (23.5)	2 (2.5)
Increased CPK	0	0	1 (33.3)	0	8 (29.6)	0	9 (23.7)	0	18 (22.2)	0
Alopecia	0	0	0	0	3 (11.1)	0	15 (39.5)	0	18 (22.2)	0
Increased ALT	3 (23.1)	0	1 (33.3)	0	5 (18.5)	0	6 (15.8)	0	15 (18.5)	0
Increased AST	3 (23.1)	0	0	0	6 (22.2)	0	6 (15.8)	0	15 (18.5)	0
Increased myoglobin blood	0	0	0	0	6 (22.2)	0	8 (21.1)	0	14 (17.3)	0
Hyponatremia	3 (23.1)	1 (7.7)	1 (33.3)	0	4 (14.8)	0	5 (13.2)	1 (2.6)	13 (16.0)	2 (2.5)
Hypomagnesemia	0	0	0	0	8 (29.6)	0	5 (13.2)	0	13 (16.0)	0
Proteinuria	4 (30.8)	0	0	0	3 (11.1)	0	4 (10.5)	0	11 (13.6)	0
Blood urine present	0	0	0	0	5 (18.5)	0	4 (10.5)	0	9 (11.1)	0
Leucopenia	2 (15.4)	0	0	0	0	0	7 (18.4)	1 (2.6)	9 (11.1)	1 (1.2)
Increased blood bilirubin	2 (15.4)	0	0	0	3 (11.1)	0	4 (10.5)	0	9 (11.1)	0

*TRAEs* treatment-related adverse events, *ALT* alanine aminotransferase, *AST* aspartate aminotransferase, *CPK* creatine phosphokinase.

SAEs related to treatment were reported in four patients (thrombosis [*n* = 1], muscular weakness [*n* = 1], and peripheral sensory neuropathy [*n* = 2]). Only one death occurred during the treatment, which was attributed to progressive disease. TRAEs led to dose reduction and treatment discontinuation in 30.9% (25/81) and 6.2% (5/81) of patients, respectively.

### PK characteristics

**Fig. 1:**
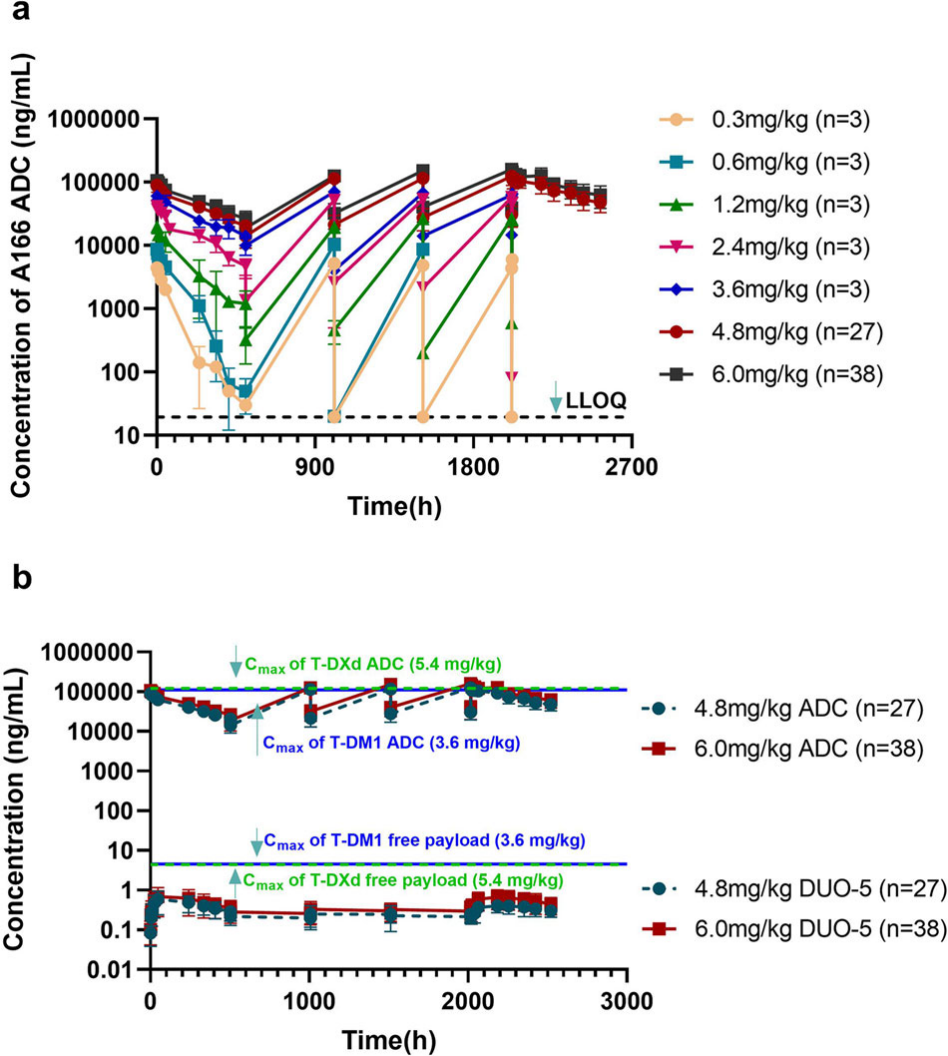
Pharmacokinetic profile of A166. **a** A166 concentration–time curve in each dose group. **b** A166 and Duo-5 concentration–time curves in the 4.8 and 6.0 mg/kg dose groups. The green dotted line indicates the mean C_max_ of T-DXd ADC and free payload in the 5.4 mg/kg dose group, and the blue solid line indicates the mean C_max_ of T-DM1 ADC and free payload in the 3.6 mg/kg dose group. Error bars indicate standard deviation (SD). Duo-5 = Duostatin-5 (anti-microtubule agent, payload of A166).

Compared with the 81-patient dataset, the PK analysis included 80 patients; one participant at a dose of 0.1 mg/kg was excluded due to a lack of PK data. Serum concentration–time profiles for the A166 ADC in cycles 1–5 at different dose cohorts are shown in Fig. 1a. Pharmacokinetic analysis of serum concentrations revealed that exposure to the A166 ADC increased with each increasing dose level and did not exhibit dose accumulation at 0.3–1.2 mg/kg, while this ADC had limited accumulation at 2.4–6.0 mg/kg. At the recommended doses for expansion (4.8 and 6.0 mg/kg), the accumulation ratio of C_max_ was approximately 1.46–1.51, and the area under the curve (AUC) was approximately 1.97–2.15 for the A166 ADC (Supplementary Table 1). The PK parameters of the A166 ADC, TA and Duo-5 for each dose cohort over cycle 1 are summarized in Supplementary Table 1. Overall, low concentrations of free Duo-5 were observed, and the PK characteristics of TA were similar to those of the A166 ADC after administration of A166. At 4.8 and 6.0 mg/kg, the t_1/2_ of the A166 ADC was 8.83 and 8.33 days after the first dose of A166, respectively. The C_max_ and AUC of Duo-5 were approximately 0.1% and 0.2% of the total A166 ADC, respectively (Fig. 1b). Furthermore, since the C_max_ of the A166 ADC at 6.0 mg/kg was approximately the same level as that of T-DXd at 5.4 mg/kg and T-DM1 at 3.6 mg/kg, the C_max_ of the free payload was approximately 9% and 13% of that of T-DXd and T-DM1 (Supplementary Table 2), respectively, indicating that A166 may have lower off-target toxicity under the same ADC exposure.

In this study, anti-A166 antibody (anti-drug antibody, ADA) was detected in 12 (14.8%) of 81 patients, 10 of whom were ADA positive at predose due to prior trastuzumab treatment. The remaining two patients tested positive in cycles 2 (before dosing) and 6 (before dosing). However, there were no differences in the exposure, safety or efficacy of the A166 ADC in ADA-positive patients compared to ADA-negative patients.

### Efficacy

In total, 80 patients were available for efficacy assessment; one patient from the 4.8 mg/kg group was not evaluated because of rapid deterioration of their general condition due to disease progression. An objective partial tumour response was observed in 43 patients. A166 showed activity at 3.6 mg/kg, and a dose‒response effect was observed with more partial responses in patients treated at 4.8 mg/kg or higher. The doses of 4.8 mg/kg and 6.0 mg/kg A166 were chosen for further investigation in the dose expansion part. At the time of the data cut-off, the median treatment duration was 6.3 months (range, 1.4–34.3) in the 4.8 mg/kg cohort and 5.4 months (range, 1.4–23.3) in the 6.0 mg/kg cohort, and the median duration of follow-up was 20.3 months (range, 1.9–34.4) in the 4.8 mg/kg cohort and 14.8 months (range, 3.1–28.4) in the 6.0 mg/kg cohort.

**Fig. 2:**
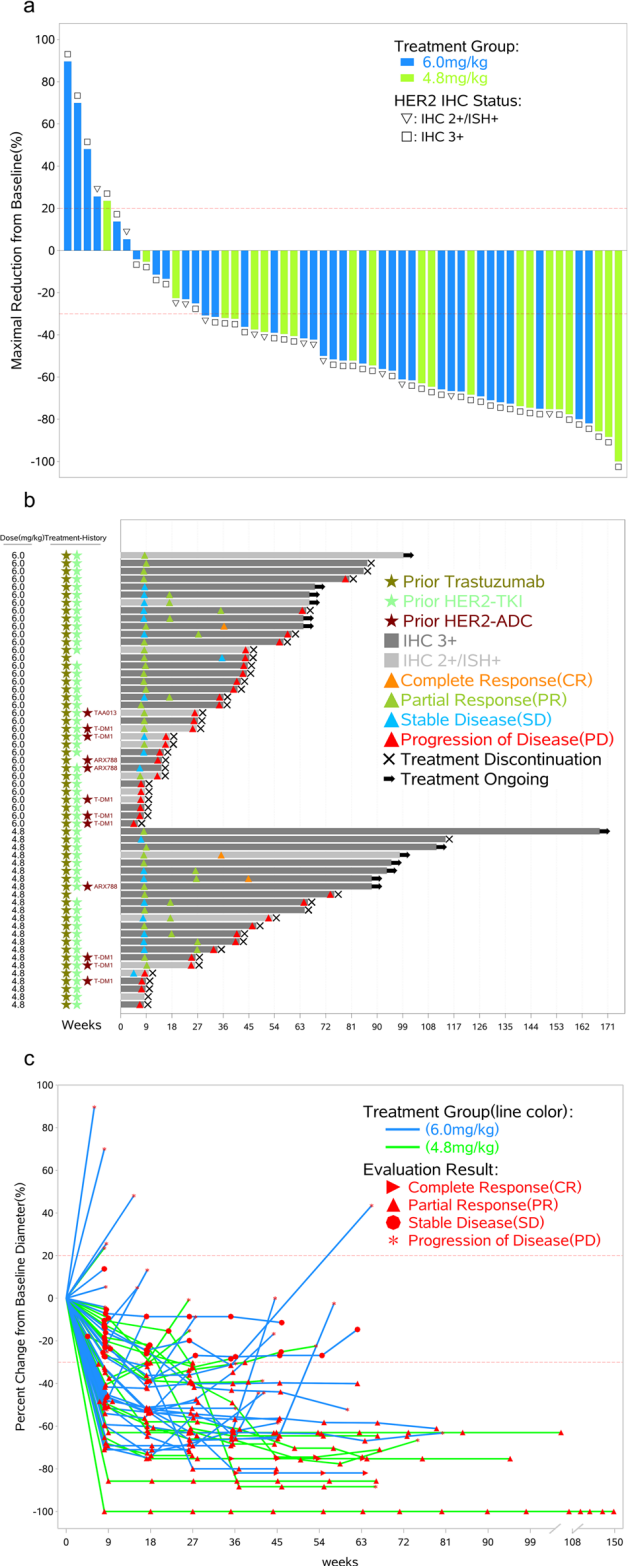
Waterfall, swimmer, and spider plots by dose level in the 4.8 and 6.0 mg/kg cohorts of HER2-positive breast cancer patients (*n* = 58). **a** Waterfall plot: maximal change in tumour target lesion size from baseline using RECIST v1.1 for patients with at least one posttreatment radiographic evaluation. The length of the bar represents the maximal decrease or minimal increase in the target lesion(s). **b** The swimmer plot shows the response and durations of response in the evaluated patients. **c** Change in individual tumour burden over time from baseline assessed using RECIST v1.1. Tumour response was assessed before treatment and once every 9 weeks until progressive disease, initiation of a new antitumour therapy, or withdrawal of consent.

In this phase I trial, for 58 HER2-positive breast cancer patients treated at 4.8 or 6.0 mg/kg (Table 3), the ORR was 70.7% (41/58; 95% CI, 57.3–81.9), and the DCR was 81.0% (47/58; 95% CI, 68.6–90.1), with all of the responding patients pretreated with trastuzumab ± pertuzumab, 39 responding patients (39/55, 70.9%) among those who had previously received at least one anti-HER2 TKI, 5 responding patients (5/12, 41.7%) among those who had previously received an anti-HER2 ADC, and 3 responding patients (3/8, 37.5%) among those who were pretreated with T-DM1. The waterfall, swimmer, and spider plots of the tumour burden alteration over time for each patient are shown in Fig. 2a–c, respectively. Seventeen of 23 patients (17/23, 73.9%; 95% CI, 51.6–89.8) achieved a response in the 4.8 mg/kg cohort, whereas 24 of 35 patients (24/35, 68.6%; 95% CI, 50.7–83.2) achieved a response in the 6.0 mg/kg cohort. The median PFS was 12.3 months (95% CI, 6.0–not reached) in the 4.8 mg/kg cohort and 9.4 months (95% CI, 4.0–10.4) in the 6.0 mg/kg cohort.

**Table 3:** Best responses for HER2-positive breast cancer patients in 4.8 and 6.0 mg/kg cohorts.

	4.8 mg/kg (*N* = 23)	6.0 mg/kg (*N* = 35)	Total (*N* = 58)
CR, *n* (%)	2 (8.7)	1 (2.9)	3 (5.2)
PR, *n* (%)	15 (65.2)	23 (65.7)	38 (65.5)
SD, *n* (%)	2 (8.7)	4 (11.4)	6 (10.3)
PD, *n* (%)	3 (13.0)	7 (20.0)	10 (17.2)
NE, *n* (%)	1 (4.3)	0	1 (1.7)
ORR (95% CI)	73.9% (51.6, 89.8)	68.6% (50.7, 83.2)	70.7% (57.3, 81.9)
DCR (95% CI)	82.6% (61.2, 95.1)	80.0% (63.1, 91.6)	81.0% (68.6, 90.1)

*CR* complete response, *PR* partial response, *SD* stable disease, *PD* progressive disease, *ORR* objective response rate (CR + PR), *DCR* disease control rate (CR + PR + SD), *CI* confidence interval.

Among patients who showed a response, one patient (4.8 mg/kg) with a diagnosis of hormone receptor-negative, HER2-positive breast cancer and lymph node metastasis showed a duration of response lasting approximately 2 years, and the treatment is still continuing. After six cycles of therapy, the CT scan revealed that the target lesion completely disappeared (Supplementary Fig. 1).

## Discussion

This was a first-in-human clinical study conducted in China to evaluate A166, a HER2-targeted ADC harbouring a microtubule inhibitor payload, in patients with advanced solid tumours (mostly [90.12%] metastatic breast cancer), who had previously received anti-HER2 therapy (trastuzumab, pertuzumab, pyrotinib, T-DM1, ARX788, etc.). The significant advantages of A166 are the high activity of the payload, which allows A166 to exhibit superior efficacy even at a relatively low DAR, and excellent stability in the circulation. The design of A166 includes the highly stable valine citrulline linker, which reduces the exposure to free payload. The C_max_ and AUC of Duo-5 were approximately 0.1% and 0.2% of those of total A166 (ADC) on a molar basis, respectively. The lower C_max_ of free Duo-5 compared with the payloads of T-DM1 and T-DXd (Supplementary Table 2) suggested that A166 was highly stable in the systemic circulation^16,17^. The toxicity of the free payload can be the dose-limiting factor, and a relatively small increase in payload exposure in the systemic circulation could lead to significant adverse effects; in contrast, the PK profile of A166 supports its low off-target toxicity, which offers patients a sustained benefit in the long term. As expected, exceptionally fewer systemic AEs often associated with chemotherapy drugs, such as gastrointestinal, haematological and pulmonary toxicities (Supplementary Table 3)^18–20^, were observed in this study for A166 compared to other approved ADCs for breast cancer, further indicating that A166 may provide an opportunity for possible combination therapy with other drug modalities. The potential for the bystander killing effect of A166 remains undefined but will be further evaluated in future studies.

The most common TRAEs with A166 were ocular AEs, which were manageable. Ocular AEs were dose-related, with one grade 2 AE in only one patient (7.7%, Table 2) when dosed at 2.4 mg/kg and lower. Fortunately, all ocular AEs were reversible and occurred approximately after cycle 2, and most were grade 1 or 2, which were easy to clinically diagnose and to assess severity through protocol-assigned eye examinations. During the trial, we followed the optimized management algorithm created by the ocular AE special task force (Supplementary Fig. 2). Patients received artificial tears prophylactically, while ocular lubricants and eyedrops with bovine serum or topical steroids were only applied during the non-DLT observation period, according to the ophthalmologist’s discretion, depending on the occurrence or grading of epitheliopathy. Treatment delay and dose reduction were used for grade 3 or 4 ocular AEs. In addition, unplanned visits were encouraged in the protocol in case ocular symptoms appeared or deteriorated. Using this strategy, A166-induced corneal epitheliopathy was generally well managed and reversible in our patients (Supplementary Fig. 3), which was further supported by a report in American patients by Sharma et al.^21^. As previously reported, ocular surface AEs occurred more often in ADCs with auristatin-F or maytansinoid DM4 as the payload (i.e., belantamab mafodotin) and conventional tubulin-binding chemotherapeutic agents (i.e., docetaxel and paclitaxel)^22–27^. In our trial, the percentages of ocular AEs of grades 1, 2, 3, 4 and 5 at the most severe level in the 4.8 mg/kg cohort were 33.3%, 22.2%, 44.4%, 0% and 0%, respectively, which is in a similar range to the corresponding 8%, 17%, 45%, 1% and 0% reported for FDA-approved 2.5 mg/kg belantamab mafodotin for 95 patients with multiple myeloma^28^. However, we did not observe any grade 4 ocular AE, corneal perforation or blindness. We are now still taking proactive measures to optimize prophylaxis, treatment and surveillance for ocular AE and exploring the complex underlying mechanisms^28–30^.

Another significant advantage of A166 was its strong antitumour activity. We found that in the 4.8 mg/kg group, A166 showed encouraging antitumour activity in heavily pretreated HER2-positive metastatic breast cancer with an ORR as high as 73.9% and a median PFS of longer than 12 months, which was equal to or higher than those of currently available HER2-directed regimens and new agents under development. Importantly, antitumour activity was also observed in the subgroup of patients who had been pretreated with T-DM1, trastuzumab, HER2-TKI, or both trastuzumab and HER2-TKI. Both ORR and PFS were numerically higher at 4.8 mg/kg than at 6.0 mg/kg (ORR, 73.9% vs. 68.6%, respectively; PFS, 12.3 vs. 9.4 months, respectively), justifying selection of the 4.8 mg/kg dose as the recommended phase II dose for the A166 pivotal phase II study (CTR20212088).

A166 has promising antitumour activity in HER2-positive breast cancer patients at 4.8 mg/kg with manageable toxicity, which led to governmental approval of a pivotal phase II registration trial in HER2-positive patients who have progressed on at least two prior lines of anti-HER2 therapies. Several ongoing studies are investigating the efficacy and safety of A166 in different HER2-expressing solid tumour types, including HER2-low breast cancer (CTR20181301), NSCLC (CTR20210516), and urothelial carcinoma (CTR20211319). These studies will enhance our understanding of A166 efficacy and safety in various settings.

## Methods

### Study design and patient selection

This was a first-in-human, open-label, multicentre, two-part, phase I study conducted in China (chinadrugtrials.org.cn CTR20181301). Patients were eligible for enrolment if they were ≥18 years of age and had histologically confirmed locally advanced/metastatic HER2-expressing (IHC ≥ 1+) solid tumours (including breast cancer) who were unable to benefit from the available standard of care, Eastern Cooperative Oncology Group (ECOG) performance status of 0 or 1, and adequate bone marrow and organ function. Patients were excluded if they had a history of intolerance to trastuzumab, symptomatic brain metastasis, or prior antitumour treatment for brain metastases within 3 months before the first study treatment. Assessment of HER2 positivity for breast cancer and colorectal cancer was performed according to the Guideline for HER2 detection in breast cancer (2014 and 2019 versions); for gastric cancer, it was performed according to the Guidelines for HER2 detection in gastric cancer (2016 version)^31–33^.

This two-part study consisted of dose escalation and dose expansion parts. Dose escalation was conducted using a standard 3 + 3 design. We selected a conservative starting dose of 0.1 mg/kg, calculated as approximately one-twelfth of the human equivalent dose (3.2 mg/kg) of the highest nonseverely toxic dose in cynomolgus monkeys (10 mg/kg). Doses were escalated up to 6.0 mg/kg. While only one patient received A166 at a dose of 0.1 as needed, three patients at each dose level were required at doses of 0.3, 0.6, 1.2, 2.4, 3.6, 4.8 and 6.0 mg/kg intravenously every 3 weeks until disease progression, withdrawal of informed consent, or intolerable toxicity. The first 21-day treatment cycle was designed for the observation of dose-limiting toxicity (DLT), which was defined as grade ≥3 nonhaematological toxicity, except for grade 3 nausea, vomiting, or diarrhoea lasting ≤3 days after optimal supportive treatment; treatment interruption for >14 days due to toxicity; grade 4 neutrophil count reduction lasting for >7 days (or >3 days after optimal supportive treatment); febrile neutropenia; grade 4 thrombocytopenia; grade 3 thrombocytopenia with bleeding; or grade 4 anaemia. Dose escalation proceeded when all three patients completed the safety evaluation at a given dose level with DLTs in less than one-third of patients. One or two feasible recommended doses (not exceeding the MTD) would be proposed for further dose expansion by discussion between the investigator and sponsor.

The study protocol and all amendments were approved by the Ethics Committee of Fudan University Shanghai Cancer Center (approval no.: 1806186-13). The study was conducted in accordance with the Declaration of Helsinki guidelines and international standards of good clinical practice. Written informed consent was obtained from all participants for the publication of their data and photographs.

### End points

The primary endpoints of this study were assessment of the safety and tolerability of A166 and identification of the maximum tolerated dose (MTD) or recommended phase II dose. Secondary endpoints included an assessment of the pharmacokinetic parameters and preliminary antitumour effect.

### Safety and antitumour effect assessments

Safety assessments included a documentation of adverse events (AEs), serious adverse events (SAEs), vital signs, clinical laboratory examination, and findings from the physical, cardiac, and ophthalmological examinations. The severity of AEs was graded according to the National Cancer Institute’s Common Terminology Criteria for Adverse Events (NCI-CTCAE; version 4.03). We established an ocular AE special task force comprising study investigators and ophthalmologists. All patients were referred to an ophthalmologist (Dr. Z.Q. Yu of The Eye and ENT Hospital of Fudan University) for baseline assessment, including ophthalmologic examinations for visual acuity, dry eye syndrome and ocular surface diseases, e.g., corneal epitheliopathy and keratitis. Patients were referred to the ophthalmologist in every cycle and at any additional time if they had ocular signs and symptoms or if the investigator deemed it necessary. All investigators were trained for the clinical grading of ocular AEs and implementation of the study algorithm. The task force determined the subsequent drug administrations in specific scenarios, such as discontinuing A166 permanently or restarting A166 at the original or lower dose, while optimizing the management algorithm to align with the patient outcomes.

The efficacy was evaluated in accordance with the Response Evaluation Criteria in Solid Tumours (RECIST) guidelines version 1.1 every 9 (±1) weeks from the first drug infusion. Patients were followed up for an additional 24 months after the last visit or until death, whichever occurred first.

### Pharmacokinetics and immunogenicity assessments

The concentrations of A166, total antibody (TA, conjugated and unconjugated), and Duo-5 (free payload) were detected using validated bioanalytical assays for pharmacokinetic (PK) studies. Plasma samples were collected at predose and postdose 0.5 (±1/4), 4 (±1/2), 8 (±1), 24 (±2), and 48 (±4) hours (h), and 8 (±1), 11 (±1), 15 (±1), and 18 (±1) days from the end of infusion in cycles 1 and 5, as well as predose and postdose 30 (±15) minutes in cycles 2–4, and at the end of treatment (EOT). In the dose expansion part, plasma samples for PK were collected at predose and postdose 30 (±15) minutes in cycles 1–5 and at EOT.

Blood samples for anti-drug antibody analyses were collected at predose in cycles 1–6, 9, 13, 17, and every eight cycles thereafter, as well as 28 days after the last dose. Antidrug antibodies to A166 were detected using electrochemiluminescence immunoassay.

### Statistical analysis

Statistical computation was performed using SAS version 9.4 (SAS Institute, Cary, NC, USA). The objective response rate (ORR) and disease control rate (DCR) with 95% confidence intervals (CIs) were calculated using the Clopper–Pearson method based on the binomial distribution. Time-to-event statistics were calculated using the Kaplan‒Meier method, and the associated confidence intervals (Cis) were calculated using the Brookmeyer-Crowley method. All statistical tests were two-tailed, with significance defined as *p* < 0.05. The PK parameters for A166, TA, and Duo-5 were calculated using noncompartmental approaches implemented in WinNonlin 8.1.

### Reporting summary

Further information on research design is available in the Nature Research Reporting Summary linked to this article.

## Supplementary information

Supplementary Information

Reporting Summary

## Data Availability

All data relevant to the manuscript are contained within the main and supplemental text. Individual participant data were deposited in the Medidata Clinical Cloud® database. The datasets used and/or analysed during the current study are available from the corresponding author on reasonable request.
