# Supplementary material for: Phase I study of A166, an antibody‒drug conjugate in advanced HER2-expressing solid tumours
Source: NPJ Breast Cancer. 2023 Apr 18;9:28. doi: 10.1038/s41523-023-00522-5 (PMC10113253; doi:10.1038/s41523-023-00522-5)
Supplement: Supplementary file 1 — Supplementary Information [file 41523_2023_522_MOESM1_ESM.pdf]

Supplementary Information

Phase I Study of A166, an Antibody–Drug Conjugate in Advanced HER2-Expressing Solid Tumours

Jian Zhang, Rujiao Liu, Shuiping Gao, Wenhua Li, Yang Chen, Yanchun Meng, Chang Liu, Wenyue Jin, Junyan Wu, Ying Wang, Yanrong Hao, Shuli Yi, Yan Qing, Junyou Ge, Xichun Hu

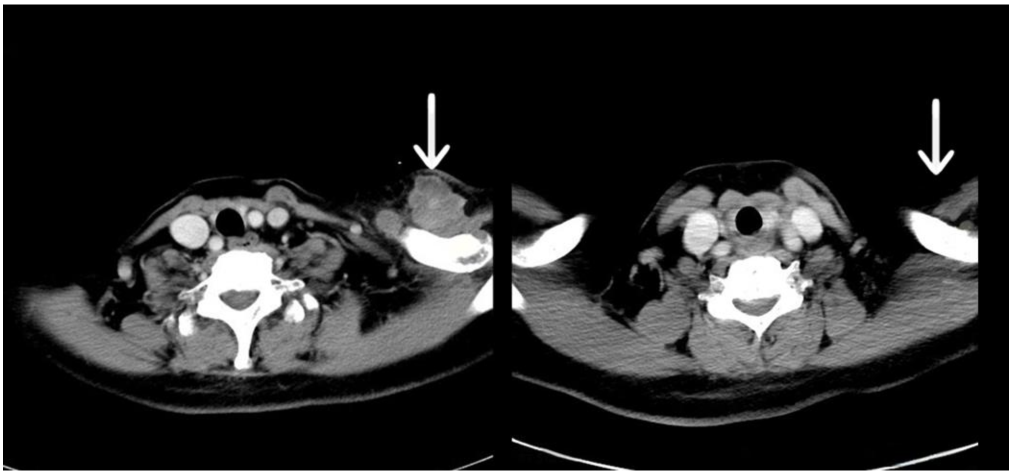

Supplementary Figure 1. One partial response with tumor shrinkage at 4.8 mg/kg after six cycles of treatment compared to baseline.

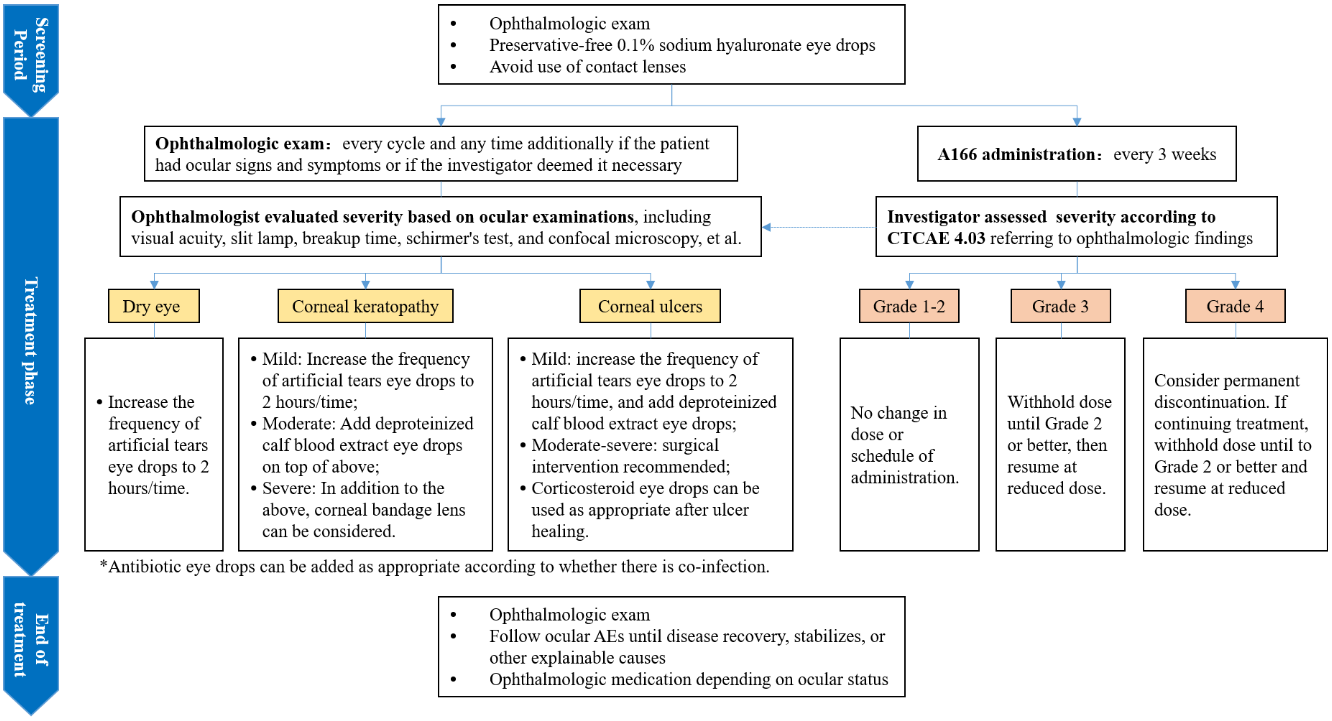

Supplementary Figure 2. The Ocular toxicity management algorithm.

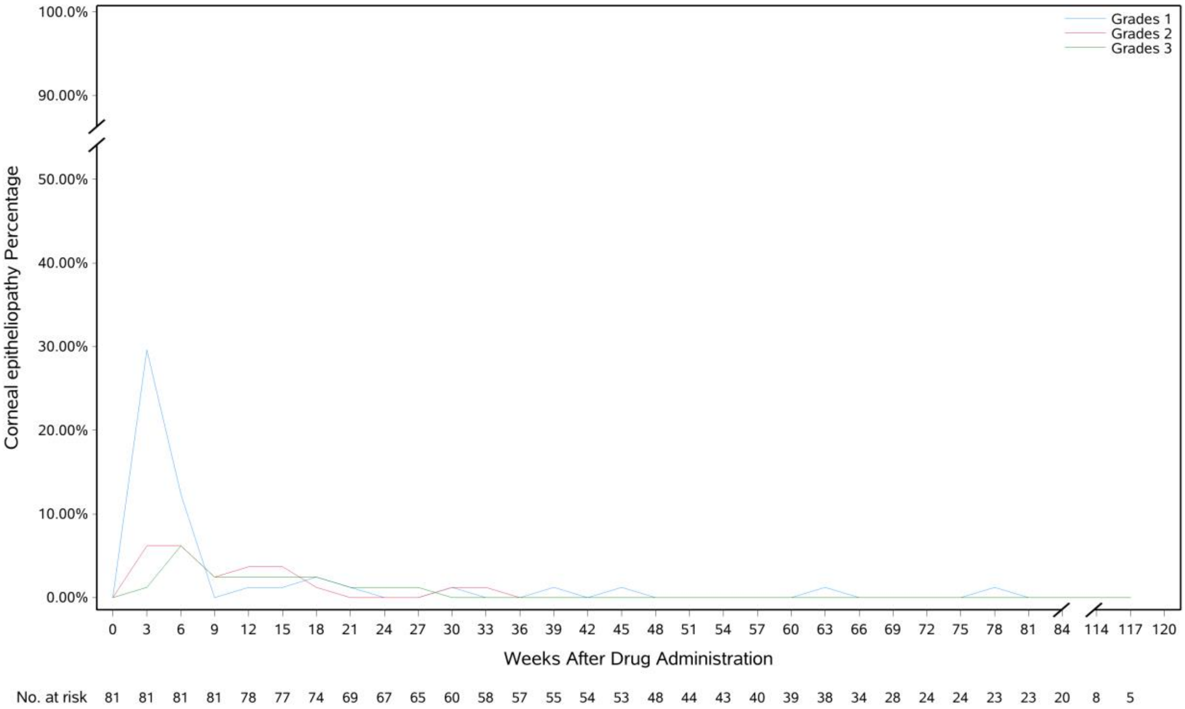

Supplementary Figure 3. Corneal epitheliopathy (%) over time. Blue, red, and green lines represent grades 1, 2, and 3, respectively, as assessed by CTCAE 4.03.

Supplementary Table 1. A) Pharmacokinetic properties of A166 ADC

	0.3 mg/kg (n=3[1])	0.6 mg/kg (n=3)	1.2 mg/kg (n=3)	2.4 mg/kg (n=3)	3.6 mg/kg (n=3)	4.8 mg/kg (n=27[2])	6.0 mg/kg (n=38[3])
Cycle 1							
C_{max} (μg/mL)	4.51 (13.0)	8.65 (14.4)	20.7 (12.4)	40.9 (15.4)	60.8 (7.6)	92.5 (20.6)	109 (17.7)
AUC_{last} (h*μg/mL)	279 (3.64)	679 (25.2)	1870 (49.8)	6390 (22.8)	12400 (23.0)	18100 (14.9)	22200 (24.2)
AUC_{inf} (h*μg/mL)	283 (3.91)	682 (25.3)	1890 (50.9)	6600 (24.8)	15000 (23.3)	22800 (18.8)	26600 (30.5)
$t_{1/2}$ (day)	1.65 (76.0)	2.06 (24.5)	2.41 (72.1)	3.68 (40.0)	7.75 (12.7)	8.83 (19.7)	8.33 (41.9)
CL (mL/h)	64.0 (11.0)	44.4 (5.97)	43.4 (52.1)	24.4 (14.8)	12.8 (27.3)	12.9 (24.5)	12.9 (29.8)
V_z (mL)	3660 (83.1)	3160 (24.3)	3620 (18.9)	3110 (38.6)	3430 (36.8)	3960 (18.0)	3720 (46.4)
Cycle 5							
R_{ac_Cmax}	1.33	NA	NA	NA	NA	1.46 (0.32)	1.51 (0.32)
$R_{ac\_AUClast}$	NA	NA	NA	NA	NA	2.09 (0.42)	1.97 (0.38)
R_{ac_AUCinf}	NA	NA	NA	NA	NA	2.15 (0.20)	2.04 (0.41)

Note:

Data in cycle 1 are mean (coefficient of variation, %); data in cycle 5 are mean (SD).

1: n=1 for R_{ac_Cmax};

2: n=27 for C_{max}; n=23 for AUC_{last}; n=20 for AUC_{inf}, $t_{1/2}$, CL, and V_z; n=14 for R_{ac_Cmax}; n=10 for $R_{ac\_AUClast}$; n=3 for R_{ac_AUCinf}.

3: n=38 for C_{max}; n=37 for AUC_{last}; n=29 for AUC_{inf}, $t_{1/2}$, CL, and V_z; n=20 for R_{ac_Cmax}; n=19 for $R_{ac\_AUClast}$; n=4 for R_{ac_AUCinf}.

Abbreviations: NA, not applicable; AUC_{inf}, area under the concentration versus time curve from time zero extrapolated to infinity; AUC_{last}, area under the concentration versus time curve from time zero to the time of the last quantifiable concentration; CL, total body clearance; C_{max}, maximum serum concentration; $t_{1/2}$, terminal elimination half-life; V_z, volume of distribution; R_{ac_Cmax}, accumulation ratio of C_{max} between cycle 1 and 5; $R_{ac\_AUClast}$, accumulation ratio of AUC_{last} between cycle 1 and 5; R_{ac_AUCinf}, accumulation ratio of AUC_{inf} between cycle 1 and 5.

Supplementary Table 1. B) Pharmacokinetic Properties of Total Antibody

	0.3mg/kg (n=3[1])	0.6mg/kg (n=3)	1.2mg/kg (n=3)	2.4mg/kg (n=3)	3.6mg/kg (n=3)	4.8mg/kg (n=27[2])	6.0mg/kg (n=38[3])
C_{max} (μg/mL)	4.97 (13.7)	9.8 (18.3)	19.5 (11.1)	43.8 (15.1)	65 (3.62)	96.6 (18.6)	112 (18.1)
AUC_{last} (μg*h/mL)	301 (15.9)	636 (30.4)	1670 (45.7)	5310 (22.4)	10100 (21.0)	15700 (13.7)	18600 (23.6)
AUC_{inf} (μg*h/mL)	304 (16.7)	645 (28.6)	1670 (45.4)	5340 (22.9)	10600 (22.7)	17100 (14.9)	20500 (27.5)
$t_{1/2}$ (day)	1.62 (146)	1.67 (15.2)	1.29 (28.3)	2.03 (34.7)	4.54 (20.4)	5.42 (23.8)	5.46 (36.1)
CL (mL/h)	59.4 (13.6)	47.0 (12.2)	49.1 (46.7)	30.1 (13.1)	18.0 (26.1)	16.9 (20.1)	16.9 (26.8)
V_z (mL)	3330 (126)	2710 (24.6)	2200 (35.0)	2120 (32.9)	2810 (11.1)	3170 (26.1)	3190 (37.0)
R_{ac_Cmax}	1.24	NA	NA	NA	NA	1.21 (0.21)	1.27 (0.25)
$R_{ac\_AUClast}$	NA	NA	NA	NA	NA	1.44 (0.25)	1.42 (0.26)
R_{ac_AUCinf}	NA	NA	NA	NA	NA	1.47 (0.22)	1.52 (0.22)

Note:
Data in cycle 1 are mean (CV %); data for R_{ac_Cmax}, $R_{ac\_AUClast}$ and R_{ac_AUCinf} are mean (SD).
1: n=1 for R_{ac_Cmax};
2: n=27 for C_{max}; n=23 for AUC_{last}, AUC_{inf}, $t_{1/2}$, CL, and V_z; n=14 for R_{ac_Cmax}; n=10 for $R_{ac\_AUClast}$ and R_{ac_AUCinf}.
3: n=38 for C_{max}; n=37 for AUC_{last}, AUC_{inf}, $t_{1/2}$, CL, and V_z; n=20 for R_{ac_Cmax}; n=19 for $R_{ac\_AUClast}$ and n=18 for R_{ac_AUCinf}.
Abbreviations: NA, not applicable; AUC_{inf}, area under the concentration versus time curve from time zero extrapolated to infinity; AUC_{last}, area under the concentration versus time curve from time zero to the time of the last quantifiable concentration; CL, total body clearance; C_{max}, maximum serum concentration; $t_{1/2}$, terminal elimination half-life; V_z, volume of distribution; R_{ac_Cmax}, accumulation ratio of C_{max} between cycle 1 and 5; $R_{ac\_AUClast}$, accumulation ratio of AUC_{last} between cycle 1 and 5; R_{ac_AUCinf}, accumulation ratio of AUC_{inf} between cycle 1 and 5.

Supplementary Table 1. C) Pharmacokinetic Properties of Duo-5

	0.3mg/kg (n=3)	0.6mg/kg (n=3)	1.2mg/kg (n=3)	2.4mg/kg (n=3)	3.6mg/kg (n=3)	4.8mg/kg (n=27[1])	6.0mg/kg (n=38[2])
C_{max} (ng/mL)	0.0876 (28.6)	0.263 (67.7)	0.348 (47.4)	0.539 (15.8)	0.272 (30.1)	0.418 (95.9)	0.603 (69.6)
AUC_{last} (ng*h/mL)	6.90 (164)	57.9 (53.7)	82.4 (50.1)	180 (48.8)	105 (28.3)	181 (46.8)	216 (50.8)
R_{ac_Cmax}	NA	NA	NA	NA	NA	1.58 (1.37)	1.04 (1.07)
$R_{ac\_AUClast}$	NA	NA	NA	NA	NA	1.27 (0.23)	1.66 (0.80)

Note:

Data are mean (CV %); data for R_{ac_Cmax}, $R_{ac\_AUClast}$ and R_{ac_AUCinf} are mean (SD).

1: n=27 for C_{max}; n=23 for AUC_{last}; n=14 for R_{ac_Cmax}; n=10 for $R_{ac\_AUClast}$.

2: n=38 for C_{max}; n=37 for AUC_{last}; n=20 for R_{ac_Cmax}; n=19 for $R_{ac\_AUClast}$.

Abbreviations: NA, not applicable; AUC_{last}, area under the concentration versus time curvefrom time zero to the time of the last quantifiable concentration; C_{max}, maximum plasma concentration; R_{ac_Cmax}, accumulation ratio of C_{max} between cycle 1 and 5; $R_{ac\_AUClast}$, accumulation ratio of AUC_{last} between cycle 1 and 5.

Supplementary Table 2. The Exposure of ADC and Free Payload of A166 Compared to T-DM1 and T-DXd

Name (dose, mg/kg)	ADC-C_{max} (nM)	free payload C_{max} (nM)	free payload/ADC (mol/mol)
A166 (4.8 mg/kg)	612.58	0.54	0.09%
A166 (6.0 mg/kg)	721.85	0.78	0.11%
T-DM1 (3.6)[19]	703.18	9.00	1.28%
T-DXd (5.4)[20]	820.00	5.97	0.73%

Abbreviations: ADC, antibody drug conjugate.

Supplementary Table 3. Comparison of Toxicity between A166 and Other ADC Drugs in Breast Cancer

TRAEs<br>No. of Patients (%)		T-DM1[21]	T-DXd[22]	Sacituzumab Govitecan[23]	A166
		N=490<br>(EMILIA)	N=184<br>(DESTINY-Breast01)	N=108<br>(Phase II)	N=65<br>(Phase I)
Dose schedule		3.6 d1, Q3W	5.4 d1, Q3W	10 d1 d8, Q3W	4.8 or 6.0 d1, Q3W
Thrombocytopenia	total	150(30.6)	39(21.2)	<10%	3(4.6)
	Grade ≥ 3	70(14.3)	8(4.3)	3(2.8)	0
Neutropenia	total	37(7.6)	64(34.8)	69 (63.9)	7(10.8)
	Grade ≥ 3	11(2.2)	38(20.7)	45 (41.7)	0
Leucopenia	total	<1%	39(21.2)	23 (21.3)	7(10.8)
	Grade ≥ 3	<1%	12(6.5)	12 (11.1)	1(1.5)
Anaemia	total	68(13.9)	55(29.9)	54 (50.0)	15(23.1)
	Grade ≥ 3	19(3.9)	16(8.7)	12 (11.1)	1(1.5)
Nausea	total	202(41.2)	143(77.7)	72(67.0)	7(10.8)
	Grade ≥ 3	4(0.8)	14(7.6)	7(6.0)	0
Vomiting	total	102(20.8)	84(45.7)	53(49.0)	4(6.2)
	Grade ≥ 3	5(1.0)	8(4.3)	7(6.0)	0
Diarrhoea	total	124(25.3)	54(29.3)	67 (62.0)	6(9.2)
	Grade ≥ 3	9(1.8)	5(2.7)	9 (8.0)	0
Interstitial lung disease	total	0	25(13.6)	0	2(3.1)
	Grade ≥ 3	0	5(2.7)	0	0

Abbreviations: ADC, antibody-drug conjugate; TRAEs, treatment related adverse events.
